# The Pressure of Fusarium Disease and Its Relation with Mycotoxins in The Wheat Grain and Malt

**DOI:** 10.3390/toxins11040198

**Published:** 2019-04-02

**Authors:** Valentina Spanic, Zvonimir Zdunic, Georg Drezner, Bojan Sarkanj

**Affiliations:** 1 Agricultural Institute Osijek, Juzno predgradje 17, HR-31000 Osijek, Croatia; zvonimir.zdunic@poljinos.hr (Z.Z.); georg.drezner@poljinos.hr (G.D.); 2 Faculty of Food Technology Osijek, Josip Juraj Strossmayer University of Osijek, Franje Kuhača 20, HR-31000 Osijek, Croatia; bsarkanj@unin.hr

**Keywords:** Fusarium, deoxynivalenol, nivalenol, zearalenone, wheat malt

## Abstract

Fusarium head blight (FHB) is one of the most destructive wheat fungal diseases, causing yield loss, quality reduction, and accumulation of mycotoxins. The aim of this research was to summarize the occurrence of major *Fusarium* mycotoxins: deoxynivalenol (DON), 3-acetyldeoxynivalenol (3-AcDON), nivalenol (NIV), and zearalenone (ZEN) in two consecutive years to search the relationship between disease incidence and severity with mycotoxins found in control and inoculated grains and corresponding malt. In addition, deoxynivalenol-3-glucoside (D3G) in one-year research was measured. Tested wheat varieties showed infection scores of 3% (‘U1’ and ‘Sirban Prolifik’) to 79% (‘Golubica’) for Type I resistance evaluation. There were few moderately resistant varieties in view of their areas under the disease progress curve, which can be considered Type III resistance (‘Sirban Prolifik’ and ‘U1’). According to the data quantified by LC–MS/MS, DON decreased in infected malt in comparison to corresponding grain, while ZEN occurred only in infected malt samples. Both 3-AcDON and NIV increased in inoculated malt in comparison to corresponding grain, due to a combination of plant metabolism and de novo synthesis by molds during malting. Based on the results, we can draw a few conclusions: the resistance to *Fusarium* decreased quantified concentrations of DON; ZEN gets synthetized during malting; unregulated 3-AcDON and NIV increase during malting; more resistant varieties have converted DON to D3G more successfully. Modified mycotoxins should be also included to legislation, since they could be transformed back to the corresponding mycotoxins under food processing conditions or during digestion.

## 1. Introduction

Fusarium head blight (FHB), caused by several *Fusarium* species, is one of the most important fungal diseases worldwide that affects the wheat (*Triticum aestivum* L.) by decreasing yield and quality [1,2]. A major concern for human and animal health is mycotoxins, whereas the most important are trichothecenes and zearalenone (ZEN) as secondary fungal metabolites. The most commonly occurring mycotoxin in many cereals and malt is deoxynivalenol (DON) [3,4], a type B trichothecene which is predominant, and regulatory limits are set for food and feed worldwide [5,6]. The general mode of action of DON is inhibition of protein synthesis and disruption of signal transmission, causing cell death [7]. Due to its high occurrence in food, DON and its metabolites are rapidly being detected in human urine [8]. The utilization of resistant wheat varieties is the best method to control FHB [9,10,11]. Different resistance mechanisms to FHB are known in wheat. The first two mechanisms of resistance include Type I: the resistance to initial infection, and Type II: the resistance for disease spread [12]. Type III and IV are related to post-harvest traits [13]: DON accumulation [14] and fusarium damaged kernels (FDK) [15], or fusarium colonized kernels (FCK) [16]. Xu (2003) concluded that environmental conditions during flowering had a higher impact on adverse effects than the conditions during the grain maturation period [17]. The correlation between FHB severity and mycotoxin accumulation is caused by the specific wheat variety resistance and weather conditions [18]. Mesterhazy (1997) [19] reported that DON levels in wheat depends on both variety resistance and isolate. In wheat, barley, and rye, the severity of symptoms on spike is often correlated with the mycotoxin accumulation in the grains [20,21,22,23,24]. However, for DON content, it was concluded that is not always highly correlated with FHB severity because those traits could be regulated by independent loci or genes [25]. Although, after detection of modified (masked) mycotoxins, the DON and its main plant metabolite deoxynivalenol-3-glucoside (D3G) were higher correlated with the FHB symptoms [26]. ZEN is more common in colder conditions in northern European countries [26], thus it is not expected in central and southern European cereals [27]. Yoshida and Nakajima (2010) [28] indicated that high levels of DON and nivalenol (NIV) can be produced by early infection where Type I resistance can be crucial in wheat defense, although the authors did not monitor modified mycotoxins, therefore the indications were incomplete. Furthermore, some *Fusarium* species, such as *F. poae*, can produce NIV and other emerging mycotoxins, such as beauvericin, enniatins, and fusarin [29], and their occurrence in cereals and malt is already documented [29]. According to Mastanjević et al. (2019) [30], the brewing industry could be affected by FHB with the negative impact on germination rates, which results in worse malting quality and malt yield reduction.

In this research, authors report the comparison of resistant, moderately resistant, and susceptible varieties of wheat to the accumulation of *Fusarium* mycotoxins during a two-year experiment, and the corresponding transfer to the malt. In addition, the occurrence of deoxynivalenol-3-glucoside (D3G) is also reported due to the metabolism of the plant, and possible conversion to the DON through the hydrolysis or de-glycosylation process in vertebrates or food processing, which contributes to the overall DON exposure [31].

## 2. Results

**Figure 1:**
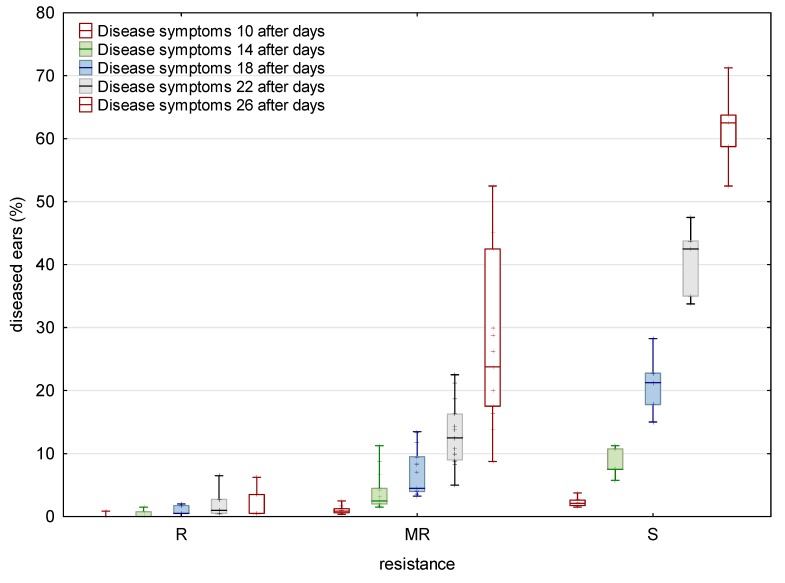
Disease symptoms for Type I resistance in tested wheat varieties after 10, 14, 18, 22, and 26 days after inoculation with *F. graminearum* and *F. culmorum* at location Osijek in 2015/2016 and 2016/2017.

**Figure 2:**
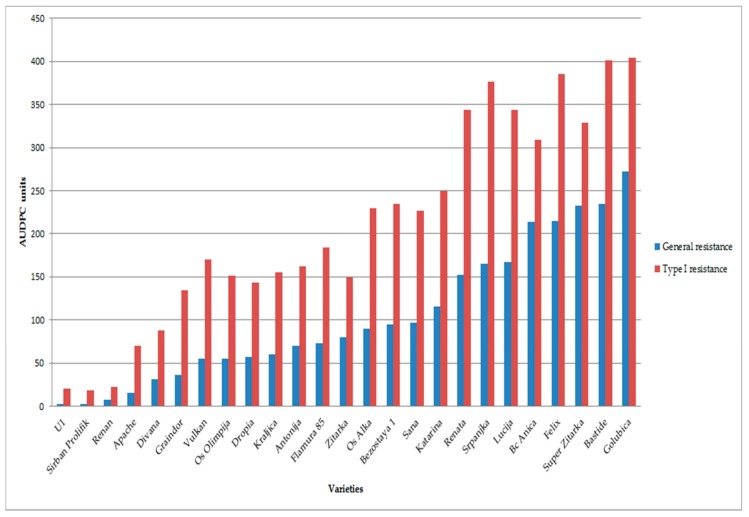
The area under disease progress curve (AUDPC) for general and initial resistance (Type I) in artificially infected treatment by *F. graminearum* and *F. culmorum* for tested wheat varieties, at location Osijek in 2015/2016 and 2016/2017.

For Type I FHB resistance, the fraction of plants showing disease symptoms ranged from 3% (‘Sirban Prolifik’ and ‘U1’) to 79% (‘Golubica’), scored at 26 days after inoculation (dai) (Figure 1). ‘Sirban Prolifik’ and ‘U1’ were the most FHB resistant varieties, while ‘Golubica’ and ‘Bastide’ were the most FHB susceptible varieties for Type I resistance, according to the calculated area under the disease progress curve (AUDPC) (Figure 2).

For the control check, the non-inoculated wheat samples were taken, where the *Fusarium* species infection rates were 0, due to the weather conditions in the given years. In the last assessment (26 dai) for general resistance varieties, ‘Golubica’ had up to 71% diseased ears (Fig 1) for Type I resistance. Two resistant varieties (‘U1’ and ‘Sirban Prolifik’) kept FHB symptoms below 1%. The range of AUDPC for general FHB resistance in the 25 tested winter wheat varieties ranged from 2.7 (‘U1’) to 272.3 (‘Golubica’) AUDPC units (Figure 2). The *Fusarium* species were morphologically determined after inoculation as *F. graminearum* and *F. culmorum* (1:1), while no other *Fusarium* species were detected.

Correlation analyses showed a statistically significant positive relationship between the amount of FHB symptoms (Type I and general resistance) and DON contamination in inoculated wheat grains (*r* = 0.91; *p* < 0.01 for Type I resistance and *r* = 0.82; *p* < 0.01 for the general resistance, respectively), FHB symptoms, and 3-acetyldeoxynivalenol (3-AcDON) contamination in inoculated wheat grains (*r* = 0.78; *p* < 0.01 for Type I resistance and *r* = 0.71; *p* < 0.01 for the general resistance, respectively), as well as between FHB symptoms and DON in the corresponding malt samples (*r* = 0.83; *p* < 0.01 and *r* = 0.77; *p* < 0.01, respectively), FHB symptoms and 3-AcDON in the corresponding malt samples (*r* = 0.79; *p* < 0.01 and *r* = 0.71; *p* < 0.01, respectively), FHB symptoms, and ZEN (*r* = 0.63; *p* < 0.01 and *r* = 0.63; *p* < 0.01, respectively) in the corresponding malt samples. FHB symptoms were statistically significantly correlated with NIV in the grain (*r* = 0.46; *p* < 0.05 and *r* = 0.43; *p* < 0.05, respectively) and in the corresponding malt (*r* = 0.48; *p* < 0.05 and *r* = 0.40; *p* < 0.05, respectively) (Table 1). Significant correlation was also obtained between DON and ZEN in infected malt (*r* = 0.67; *p* < 0.01), DON, and 3-AcDON in infected malt (*r* = 0.85; *p* < 0.01), and DON and NIV in infected malt (*r* = 0.67; *p* < 0.01). In infected malt, 3-AcDON showed a statistically high significant correlation with ZEN (*r* = 0.63; *p* < 0.01) and a statistically significant correlation with NIV (*r* = 0.47; *p* < 0.05) (Table 1).

High positive statistically significant correlations were observed between DON in infected grain and corresponding infected malt (*r* = 0.85; *p* < 0.01), and between 3-AcDON in infected grain and corresponding infected malt (*r* = 0.79; *p* < 0.01) (Table 1).

**Figure 3:**
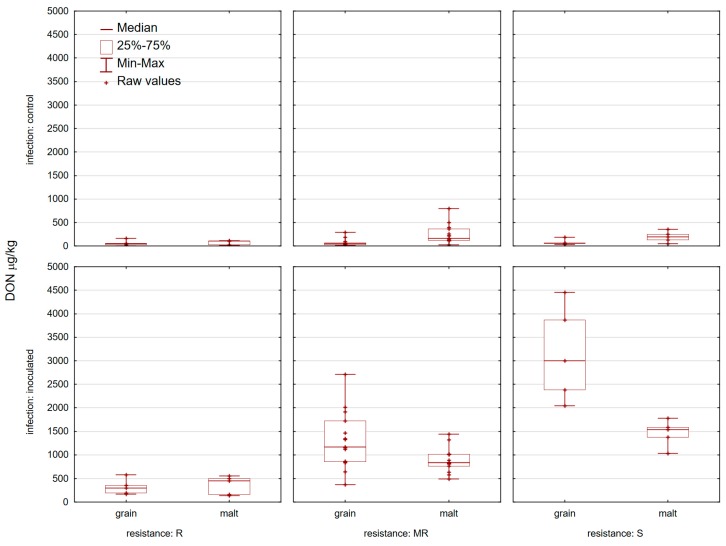
Deoxynivalenol in 25 control and inoculated wheat grain and corresponding malt samples at location Osijek in 2015/2016 and 2016/2017.

**Figure 4:**
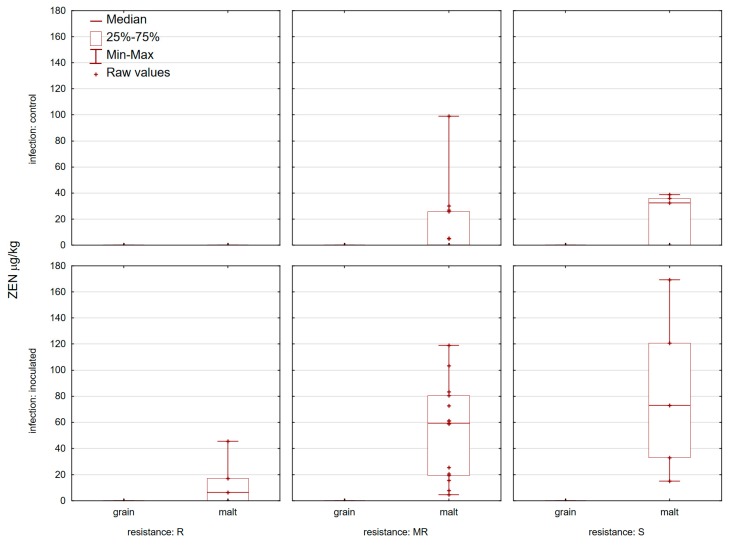
Zearalenone in 25 control and inoculated wheat grain and corresponding malt samples at location Osijek in 2015/2016 and 2016/2017.

**Figure 5:**
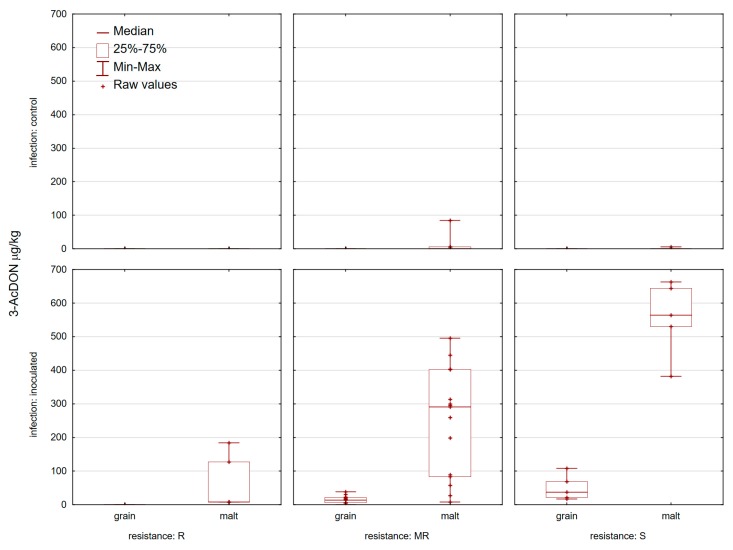
3-acetyldeoxynivalenol (3-AcDON) in 25 control and inoculated wheat grain and corresponding malt samples at location Osijek in 2015/2016 and 2016/2017.

**Figure 6:**
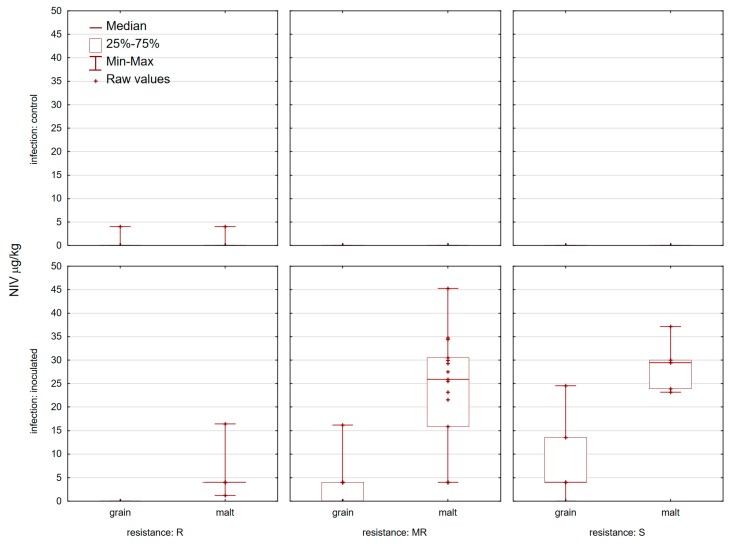
Nivalenol (NIV) in 25 control and inoculated wheat grain and corresponding malt samples at location Osijek in 2015/2016 and 2016/2017.

None of the control samples analyzed contained mycotoxins (DON and ZEN) at or above the EU maximum permitted levels set out in commission regulation (EC) No. 1881/2006 for unprocessed wheat (Figure 3 and Figure 4). Control grain samples contained 19 samples with 3-AcDON and only one sample with NIV (Figure 5 and Figure 6). DON was present in 25/25 control wheat grain samples, with an average level of 70.1 µg/kg and in all corresponding malt samples (201.5 µg/kg). In 15 samples of infected grain DON levels were >1000 µg/kg with an average level of 1474 µg/kg. The highest amount of DON was detected in ‘Bastide’ (4457.8 µg/kg) and the lowest one was detected in ‘Renan’ (173.4 µg/kg) (Figure 3). In general, malt samples which were inoculated contained less DON than the samples from which they were prepared (the exceptions were five varieties: ‘Graindor’, ‘Apache’, ‘U1’, ‘Os Alka’, and ‘Bezostaya 1’) (Figure 3). 3-AcDON was found in all inoculated wheat samples after malting, with levels ranging from 8.3 µg/kg (‘Sirban Prolifik’, ‘Renan’, ‘Apache’, and ‘Os Olimpija’) to 662.7 µg/kg (‘Bastide’) (Figure 5). Instead of a DON decrease in infected malt in comparison to grain, an increase of ZEN content was observed in the malted wheat in comparison with the raw material (Figure 4). In contrast to infected grain samples, inoculated malt samples contained ZEN. In total, 23 of the 25 inoculated malt samples contained ZEN, at concentrations ranging from 0 (‘Sirban Prolifik’ and ‘Renan’) to 169.3 μg/kg (‘Super Zitarka’). For ZEN (mean 50.9 μg/kg), four samples exceeded the respective limit (100 μg/kg), and the mean content of NIV was 21.2 μg/kg (Figure 6). In control and inoculated grains, there was no ZEN (Figure 4). 

**Figure 7:**
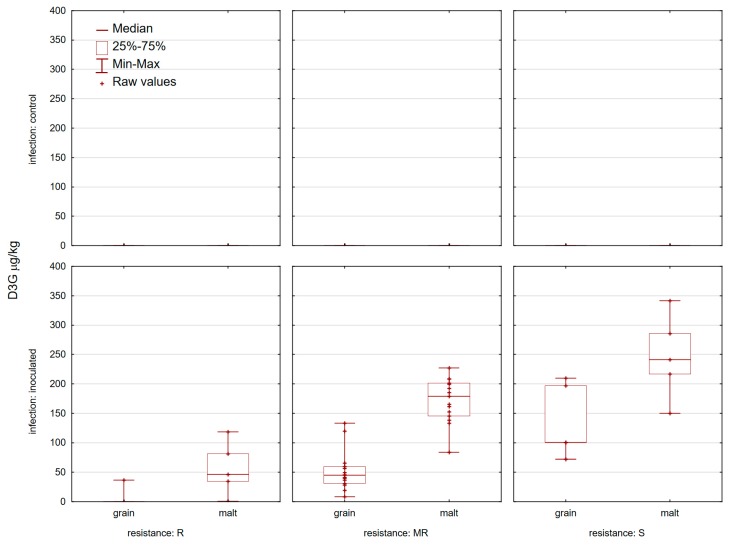
Deoxynivalenol-3-glucoside in 25 inoculated wheat grain and corresponding malt samples at location Osijek in 2016/2017.

D3G mycotoxin in naturally-contaminated (control) crops was below the limit of detection in wheat samples before and after malting (Figure 7). This mycotoxin was the second most abundant mycotoxin in the infected grain, found in 21 of the 25 winter wheat varieties with levels ranging from 8.55–210 µg/kg, with an average level of 59.9 µg/kg. ‘Sirban Prolifik’, ‘Renan’, ‘U1’, and ‘Apache’ had no D3G in wheat infected grains. The highest level of D3G was found in a sample which was highly infected with FHB (‘Golubica’, 210 µg/kg). However, none of the tested varieties have proved fully immune after malting. D3G was present in all winter wheat varieties after malting of inoculated samples with the range 0.71–341.4 µg/kg, with an average value of 163.9 µg/kg. A lower amount of D3G content (<100 µg/kg) was detected in infected malt samples in ‘Sirban Prolifik’, ‘Renan’, ‘U1’, ‘Divana’, and ‘Graindor’. After malting, ‘Golubica’ showed the highest value of D3G (Figure 7).

## 3. Discussion

### 3.1. Relationship of Toxin Measurement to Resistance

Twenty-five winter wheat varieties were evaluated in the field conditions for two components of FHB resistance during two consecutive years. The evaluation of Type I and general FHB resistance was carried out using spray inoculation with a mixture of *F. graminearum* and *F. culmorum* (1:1) and measuring disease incidence and percentage of infected heads in the plots, which is a standard method for FHB evaluations [9]. All winter wheat varieties developed disease symptoms with a wide range of resistance or susceptibility. It took 10–18 days for symptoms to show up. The time required to develop FHB symptoms was longer at more resistant varieties than at susceptible ones, which is in accordance with the previous research of Bai and Shaner (1996) [32]. Most varieties started to develop symptoms at 10 dai (except the three resistant one) when they started to increase. The FHB resistance of some varieties (‘Sirban Prolifik’ and ‘U1’) is in accordance with previous research, where those varieties were declared as resistant [11,16]. Along with these resistant varieties, ‘Apache’, ‘Renan’, and ‘Divana’ also showed lower FHB initial infection, indicating that resistance to initial infection was higher. ‘Graindor’, ‘Dropia’, ‘Zitarka’, ‘Os Olimpija’, and ‘Kraljica’ had relatively higher AUDPC (<160) for initial infection and, compared to resistant varieties, they were regarded as moderately resistant. Our disease scoring was similar to the research of Palacios et al. (2011) [33], where 55 durum wheat (*Triticum turgidum* L. var. *durum*) samples were collected during two harvest seasons (2007 and 2008) showing *Fusarium* contamination, with infection levels ranging from 8 to 66%. For control check, the non-inoculated plots were taken, where the infection rate of *Fusarium* were 0 due to climate conditions in given years. In the last assessment (26 dai) for general resistance varieties, ‘Golubica’ had up to 71% *Fusarium* symptoms, similar to Type I resistance. Similar data for resistance was obtained both for Type I and general resistance. 

FHB severity and mycotoxins in the grain (DON, 3-AcDON, NIV) and the malt (DON, 3-AcDON, ZEN, NIV) were highly correlated. Chrpová et al. (2007) [34] and Lemmens et al. (2016) [35] concluded on the highly significant relationship between the amount of FHB symptoms and DON contamination where DON-resistant varieties were also *Fusarium* resistant. It is believed that breeding for resistance to FHB will increase resistance to DON accumulation in most cases, as confirmed by our research, which gives the significance to post-harvest mycotoxin testing. 

A highly positive relationship between DON in the grain and other mycotoxins in the grain and malt suggests that samples with high DON levels would likely produce malt with high mycotoxin levels, so resistant varieties should be a major safety factor in malt/beer production. Similarly, this was concluded by Schwarz et al. (2006) [36] and Váňová et al. (2004) [37] for barley samples. Overall, there was a significant and positive correlation between DON and ZEN, similarly as it was obtained by Duffeck et al. (2017) [38]. Also, according to one year of data, DON and D3G were highly correlated. This was in accordance with Lemmens et al. (2016) [35], who revealed a highly significant relationship between the amount of FHB symptoms and DON contamination and concluded that the reduction of DON content will reduce D3G content. 

In the present study, ‘U1’, ‘Sirban Prolifik’, ‘Apache’, and ‘Renan’ exhibited the highest level of general resistance and appeared to possess both Type I and Type III resistance due to lower amounts of DON in the infected grains, which was potentially a stimulation of higher epidemiological conditions. The same varieties had no D3G in the infected grain samples in the 2016/2017 vegetation season. Conversely, a few varieties (‘Vulkan’, ‘Os Olimpija’, ‘Dropia’, and ‘Kraljica’) with moderate levels of FHB resistance in the field trials were susceptible to DON accumulation, indicating that their resistance was predominantly Type I and general, but was without Type III resistance. In our investigation, DON was found in a surprisingly low fraction on average in control, compared to the fraction of inoculated wheat samples, but the natural infection (control) depends mainly on the previous crops and the weather conditions during flowering of the wheat [35]. A similar trend was confirmed by Bryla et al. (2018) [39].

### 3.2. Development of Mycotoxins in the Wheat Grain and Malt

The maximum content of DON in unprocessed wheat, as defined in EU regulations (1250 µg/kg), was exceeded in 12 inoculated grain samples and in 6 inoculated malt samples. There was elevation of DON in all corresponding malt samples in comparison to the grain. In natural infection (control samples) there was an average level of DON 70.1 µg/kg. Higher mycotoxin infection was recorded in the research of Vrabcheva et al. (1996) [40] with the average level of DON 180 μg/kg and a maximum concentration of 1800 µg/kg in natural occurrence, which was very much characterized due to heavy rainfall in the spring and summer months. In Romanian feeding stuff, the median value for DON in 25 wheat samples was 880 μg/kg [41]. Furthermore, Brera et al. (2013) [42] analyzed 472 pasta samples where the mean DON contamination was 64.8 μg/kg.

The reduction of DON in malt samples, in comparison to grain, indicates that DON content can be reduced or transformed during the commercial malting process, and there is even a possibility that, during steeping, the level of DON can sometimes decrease and is no longer detectable [43]. The decrease of DON in malt in comparison to grain was in accordance with preliminary research of Spanic et al. (2018) [11]. Indeed, malting had a strong effect on mycotoxin content: in particular, a large decrease in the content of DON was observed, a phenomenon partially explained by the dissolution in the malting water, while some parts of DON could be metabolized to D3G according to one year of research. This can be attributed to relatively dry weather, because crucial conditions for mycotoxin production by *Fusarium* species include humidity, temperature, aerate, and substrate type [44]. 

The presence of DON and ZEN derivatives can be a risk to food safety. Lorenz et al. (2019) [45] concluded that modified forms of mycotoxins present an ideal case study for the critical evaluation of modified mycotoxins in food safety. After malting, inoculated samples increased NIV. According to Abbas et al. (2013) [46], NIV is more toxic than DON towards animals, while DON is more toxic towards plants. The observation of mycotoxins increment after malting is in accordance with the research of Habler et al. (2018) [47], who found in barley, after malting, an increase of the type B trichothecenes.

ZEN was detected only in infected malt samples, which can be explained by the fact that contamination of grain is largely restricted to the outer layers of wheat grain, and therefore is partitioned after malting and by the large dependents on humidity and temperatures. This could result in higher concentrations of ZEN in malt products. In the research of Galaverna et al. (2009), a high increase of ZEN content was observed in the malted wheat in comparison with the raw material [48]. Similar results of ZEN contamination of infected malt samples have only been documented before by Spanic et al. (2018) [11]. The ZEN producer *F. graminearum* prefers higher temperatures and high water activity for ZEN production, and such conditions are met during the malting. However, during favorable weather conditions, ZEN will also occur in grain wheat grain samples. According to Edwards (2011) [49], more than 20% of wheat grain samples in the UK, due to delayed wet harvest, exceeded the European limit for unprocessed cereals of 100 µg/kg and, consequently, in high fiber cereal products. In contradiction to our research on wheat grain, Piacentini et al. (2018) [50] detected much higher concentration of ZEN in brewing barley varieties that naturally occurred in the grains. These differences could be according to different *Fusarium* species occurring at different climatic areas or different weather conditions in the year when research was done. 

It is important to track the accumulation of masked mycotoxin, because, for example, D3G can represent a possible hazard to human and animal health. [51]. The determination of D3G has been done in one year of research (2016/2017) and for this reason mycotoxin was introduced later in the manuscript as a tool for fulfilling a broader image about mycotoxins during a *Fusarium* attack and its relationship with other mycotoxins before and after malting. D3G was present in all winter wheat varieties after malting of inoculated samples. Berthiller et al. (2005) [52] found that D3G accumulation was in higher ranges in inoculated samples in comparison to our study. Based on one year of data, we can conclude that more resistant varieties converted DON to D3G more rapidly, which is less toxic then DON. This is in accordance with previous research, where it was concluded that DON-resistant wheat varieties were more efficient in the conversion of DON to D3G than more susceptible varieties [53]. In the research of Maul et al. (2012) [54], 50% DON conversion occurred after five days of germination in grain cereals during malting. We can conclude that DON was converted into glycosylated form during malting as a result of the increased enzymatic activities. Similar results were obtained for barley, where final malt contained approximately 80% of DON and 260% of D3G levels compared to the initial barley [55]. Lemmens et al. (2016) [35] got similar results on wheat to those in our research. In the research of Trombete et al. (2016) [56], the presence of D3G occurred simultaneously with DON in 100% of the D3G positive samples. Furthermore, in this investigation we detected modified mycotoxin (D3G) in 84% of the grain infected samples and in 100% of the malt infected samples. Some authors found samples with D3G in flours, breakfast cereals, and snacks from the Czech market [57]. Also, it has been shown that D3G levels increase after malting of barley grains and the contaminant is transferred into beer [58]. In the previous research of Peters et al. (2017) [59] the major mycotoxins detected in the beer were DON and its plant metabolite (D3G).

## 4. Conclusions

In conclusion, our results suggest that (1) resistance to Fusarium decreases the median of quantified concentrations of DON; (2) ZEN gets synthetized during malting; (3) NIV will increase during malting; (4) more resistant varieties will convert DON to D3G more successfully. 

## 5. Materials and Methods

### 5.1. Inoculum Production 

Spore cultures of *Fusarium graminearum* (PIO 31, isolate from the field of eastern Croatia obtained from a single spore technique) and *F. culmorum* (IFA 104, DON chemotype and highly aggressive, which was obtained from the Institute of Biotechnology, IFA-Tulln, Austria) were sub-cultured on synthetic nutrient-poor agar (SNA) medium. After growing 10 days on SNA, the agar was cut into plugs and these were used for multiplication of spores in a liquid mung bean medium by the bubble breeding method [60], or in a mixture of wheat and oat grains (3:1 by volume) [61]. The conidia of both *Fusarium* species (1:1) were counted on a hemocytometer and suspensions were diluted or concentrated to reach the concentration of 1 × 10^5^ mL^−1^. The spore suspensions were set to a concentration so that a single bottle of one strain contained a sufficient amount of suspension (>900 mL), which could be diluted in 100 L of water right before inoculation (100 mL per m^2^).

### 5.2. Field Trials

The study was conducted at the Agricultural Institute Osijek in the east part of Croatia (45°32′ N, 18°44′ E) in 2015/2016 and 2016/2017. In this site, soil is eutric cambisol with a pre-crop of soybean in both seasons. The standard fertilization (NPK) was applied. Meteorological data for vegetation seasons in Osijek were obtained from the Croatian Meteorological and Hydrological Service. The obtained climate data include average monthly precipitation and temperatures in 2015/5016 and 2016/2017. The sum of precipitation in the vegetation season was 595 and 482 mm, respectively, with average temperatures of 9.7 and 10.0 °C, respectively. The two treatments (control and Fusarium inoculated) were arranged in a randomized block design with two replications for each treatment. The area of each plot was 7.56 m^2^.

The inoculation was carried out at the time of flowering for twenty-five winter wheat varieties (Zadok’s scale 65) and repeated two days later [62] (Table 2). The plots were irrigated with tractor back-sprayer on several occasions during the day to maintain humidity on the ears. The control group consisted of plants exposed to natural disease infection without water treatment. No fungicide or plant growth regulators were used in this trial. Visual scoring started on the 10th day after inoculation and was repeated the 14th, 18th, 22nd, and 26th day after inoculation by assessing two parameters (general resistance and Type I resistance). General resistance (percentage of diseased spikelets in the plot) was estimated according to a linear scale (0–100%). Type I resistance represented a percentage of diseased ears per plot after assessing a random sample of 30 heads. With this data, the area under disease progress curve (AUDPC) for general and Type I resistance was calculated for each entry.

### 5.3. Malting

The wheat grains were dried to content below 14% and stored in the dark at 20 °C for six weeks to overcome seed dormancy. Afterwards, exactly 200 g of each variety was malted in an automated Joe White malting systems micro-malting unit (Perth, Australia) at the Agricultural Institute Osijek, Croatia. Samples were placed randomly within the micro-malting unit. The malting program consisted of a 37 h interrupted steep program (16 °C, 5 h submerged, 17 °C, 12 h air rest with 100% airflow, 17 °C, 6 h submerged, 18 °C, 12 h air rest with 100% airflow, 17 °C, 2 h submerged), a 96 h germination program (17 °C, 75% airflow, 1.5 turn every 2 h), and a 18 h kilning program (60 °C, 6 h; 65 °C, 3 h; 68 °C, 2 h; 70 °C, 2 h; 80 °C, 2 h; 83 °C, 2 h; 85 °C, 1 h). The moisture decreased from 14% moisture wheat to 4–6% moisture malt. Rootlets and seedlings were removed, and the finished malt was then stored in plastic containers with caps at –20 °C until mycotoxin analysis. 

### 5.4. Mycotoxin Analyses

Mycotoxin analysis on all samples was performed by LC-MS/MS according to Ren et al. (2007) [63]. DON and its acetylated form 3-AcDON, NIV, and ZEN were extracted from grinded samples (10 g) by SPE columns (as described in Spanic et al. (2018)), and part of the extract was purified by immunoaffinity columns (IAC) for D3G isolation [64]. The LC/MS/MS analysis was performed on Perkin Elmer Series 2000 binary pump auto sampler, coupled with API2000 Triplequadrupole MS (SCIEX). The Ascentis Express C-18 column 150 × 2.1 mm; with 2.7 µm particle size, and Ascentis Express guard column (0.5 cm × 2.1 mm, with 2.7 µm particle size. The flow rate was set to 200 μL/min and the injection volume was 10 μL. Eluent A was 10 mM formic acid, eluent B was methanol with 10 mM formic acid. The quantitative determination of all compounds was performed by operating the mass spectrometer in two ESI runs (positive and negative ionization modes). Results were analyzed with Analyst software version 1.4.2. 

### 5.5. Method Validation

The used method vas validated and fit for purpose. Some validation results were included in a previous publication [11]. Concentrations for each mycotoxin were approximately: 5, 10, 50, 100, 200, 500, and 1000 µL min^−1^ (the exact concentration may vary 2% depending on the exact concentration in the purchased mix), except for D3G, where the concentration range was: 5, 10, 20, 25, 50, 75, and 100 µL min^−1^. Validation parameters are summed up in Table 3.

### 5.6. Statistical Analysis

To estimate disease progress, the AUDPC was used to combine multiple observations from five data points (different dates) into a single value [65]. For correlation analyses, Spearman’s coefficient was applied. Statistical analysis was performed with Statistica 13 (Dell software). The normality of the data distribution was tested by the Shapiro–Wilks test, the homoscedasticity was tested by using Levene’s test, the differences between multiple impendent variables were tested by the Kruskal–Wallis test, and the exact differences between the two groups were tested by the Mann–Whitney test, since the data were not normally distributed.

## Figures and Tables

**Table 1:** Correlation analysis between mycotoxin accumulation and the area under the disease progress curve (AUDPC) for general and Type I (initial) resistance during two consecutive years.

	DON-grain	3-AcDON-grain	NIV-grain	DON-malt	3-AcDON-malt	ZEN-malt	NIV-malt	AUDPC-GR	AUDPC-IN
DON-grain	1								
3-AcDON-grain	0.8239 **	1							
NIV-grain	0.4879 *	0.5570 **	1						
DON-malt	0.8462 **	0.8597 **	0.6138 **	1					
3-AcDON-malt	0.7981 **	0.7883 **	0.5153 **	0.8451 **	1				
ZEN-malt	0.5513 **	0.5054 **	0.6491 **	0.6682 **	0.6337 **	1			
NIV-malt	0.4992 **	0.6007 **	0.5214 **	0.6661 **	0.4709 *	0.5743 **	1		
AUDPC-GR	0.9085 **	0.7786 **	0.4561 *	0.8339 **	0.7942 **	0.6328 **	0.4745 *	1	
AUDPC-IN	0.8185 **	0.7073 **	0.4289 *	0.7662 **	0.7087 **	0.6309 **	0.3962 *	0.9585 **	1

**—significant at 0.01; *—significant at 0.05; GR—general resistance; IN—initial (Type I) resistance

**Table 2:** Origin and year of release of 25 investigated winter wheat varieties.

Varieties	Origin	Year of Release
U1	HR, AIO	1936
APACHE	FRA	1998
SIRBAN PROLIFIC	HU	1905
RENAN	FRA	1991
DIVANA	HR, JS	1995
GRAINDOR	FRA	2006
OS OLIMPIJA	HR, AIO	2009
BEZOSTAYA-1	Former USSR	1955
FLAMURA 85	ROM	1989
KRALJICA	HR, AIO	2010
VULKAN	HR, AIO	2009
DROPIA	ROM	2006
ZITARKA	HR, AIO	1985
ANTONIJA	HR, AIO	2011
SANA	HR, BC	1983
LUCIJA	HR, AIO	2001
OS ALKA	HR, AIO	2003
KATARINA	HR, AIO	2006
SRPANJKA	HR, AIO	1989
BC ANICA	HR, BC	2010
GOLUBICA	HR, AIO	1997
RENATA	HR, AIO	2006
BASTIDE	FRA	2003
FELIX	HR, AIO	2007
SUPER ZITARKA	HR, AIO	1997

AIO—Agricultural Institute Osijek, JS—Jost sjeme, BC—BC Institute.

**Table 3:** Validation parameters of the used LC-MS/MS method for mycotoxins quantification.

Analyte	Polarity	Retention Time (min)	*R_E_* (%)	*R_A_* (%)	*SSE* (%)	RSD Interday	RSD Intraday	LOD Matrix (ng g^−1^)	LOQ Matrix (ng g^−1^)
NIV	−	2.4	81	70	70	5.3%	9.6%	6.1	21.0
DON	−	2.5	102	101	99	6.0%	10.4%	4.8	15.1
D3G	−	2.4	88	68	78	8.2%	13.5%	5.2	15.2
3-AcDON	+	3.1	92	88	96	5.1%	7.1%	5.15	16.2
ZEN	−	19.8	73	82	109	11.2%	13.6%	1.2	3.1

RE—extraction efficiency; RA—apparent recovery; SSE—signal suppression/enhancement; RSD—relative standard deviation; LOD—limit of detection; LOQ—limit of quantification.
